# The frequency of acceptance of oral glucose tolerance test in Turkish pregnant women: A single tertiary center results

**DOI:** 10.14744/nci.2021.80588

**Published:** 2022-04-13

**Authors:** Havva Sezer, Dilek Yazici, Hande Bulut Canbaz, Mehmet Gokhan Gonenli, Aslihan Yerlikaya, Baris Ata, Bahar Bekdemir, Emine Ayca Nalbantoglu

**Affiliations:** 1 Department of Endocrinology and Metabolism, Koc University Faculty of Medicine, Istanbul, Turkey; 2 Koc University Faculty of Nursing, Istanbul, Turkey; 3 Department of Internal Diseases, Koc University Faculty of Medicine, Istanbul, Turkey; 4 Department of Internal Diseases, Yale New Haven Health Bridgeport Hospital, USA; 5 Department of Obstetrics and Gynecology, Koc University Faculty of Medicine, Istanbul, Turkey

**Keywords:** Acceptability, frequency, gestational diabetes mellitus, oral glucose tolerance test

## Abstract

**Objective:**

It is thought that there is not enough data about the frequency of acceptance of oral glucose tolerance test (OGTT) in Turkish pregnant women. The aim of this study was to investigate the frequency of acceptance of OGTT among participants in our single tertiary center.

**Methods:**

The data of non-diabetic 344 pregnant women seen at the Obstetrics Clinic of our hospital between September 2016 and September 2017 were obtained from the hospital records. Women who did not have regular follow-up during pregnancy were excluded. One of the two or one-step approaches was used in the diagnosis of gestational diabetes mellitus (GDM) depending on the choice of the physician following the patient.

**Results:**

There were 223 subjects eligible for the study. One hundred seventy-seven pregnant women (79.4%) accepted to do OGTT. We determined that 46 women (20.6%) did not complete at least one OGTT, of whom 74% (n=34) never completed the recommended screening test in this cohort. The overall frequency of GDM was approximately 15.2% (n=34). OGTT acceptability was higher among pregnant women with university graduates (p=0.02). Adverse pregnancy outcomes were similar between the accepted and rejected groups. Among the reasons for OGTT rejection, the media had a significant influence (n=35).

**Conclusion:**

Our results show that a significant percentage of patients refused to do OGTT. Therefore the actual frequency of pregnant women with GDM could not be determined. One way to increase compliance may be recommending only the one-step test for pregnant women in countries with a high rejection rate of OGTT.

**G**estational diabetes mellitus (GDM) is defined as carbohydrate intolerance with onset or 1st time detected during pregnancy in people who have no previous history [1]. The first description of GDM was made in 1828, when a woman was diagnosed with diabetes during pregnancy, which resolved after delivery [2]. GDM is associated with increased fetal and maternal mortality and morbidity [1]. Mothers with GDM are at increased risk of developing future type 2 diabetes mellitus, cardiovascular diseases, gestational hypertension, pre-eclampsia, polyhydramnios, and cesarean section [1, 3]. Babies of mothers diagnosed with GDM are at increased risk of macrosomia, birth trauma, and neonatal metabolic complications (such as hypocalcemia and hypoglycemia) [1, 3]. In addition, children of women with a history of GDM are more prone to be obese, have glucose intolerance, and diabetes in childhood or adulthood [1, 3]. The early diagnosis, treatment, and close monitoring of GDM are significantly important since GDM is related to these perinatal, neonatal, and maternal complications. The risk factors for GDM are high-risk ethnic groups, high maternal age, family history of diabetes in first-degree relatives, previous history of GDM, excessive weight gain in pregnancy, prepregnancy obesity, polycystic ovarian syndrome (PCOS), maternal history of hypertension, and previous history of macrosomic fetus [4].

The gold standard test for diagnosis of GDM is oral glucose tolerance test (OGTT). Pregnant women usually undergo screening for GDM between the 24^th^ and 28^th^ gestational week, if they are not at high risk of developing GDM. If the pregnant woman has risk factors for GDM, screening should be performed at the first antenatal visit. There is controversy to what is the best approach and diagnostic criteria for GDM screening and diagnosis. International Association of Diabetes Pregnancy Study group (IADPSG) and World Health Organization (WHO) recommend the one-step approach with 75 g 2 hour (h) OGTT and new diagnostic criteria based on data from Hyperglycemia and Adverse Pregnancy Outcome study. American Diabetes Association recommends the two-step approach with 50 g 1 h followed for positive test by the 3 h 100 g test using Carpenter and Coustan (C and C) criteria [5–7]. In Turkey, National Endocrinology and Metabolism Society recommend to use either of the two approaches [8]. To use one-step approach for screening GDM is increasing gradually but most of the clinics prefer to use two-step approach in our country.

The prevalence of GDM is increasing in many countries and in Turkey [9–11]. There are many studies about the prevalence of GDM worldwide [10–14]. In our country, there are regional studies in this regard [15, 16]. It is known that the prevalence of GDM varies among countries and different regions of the world. The cause of the high heterogeneity in prevalence may be due to variability among racial, ethnic, demographic, sociocultural, and economic factors. Furthermore using different diagnostic criteria and screening methods by different countries may be other reasons [17–19].

**Highlight key points**

• The frequency of acceptance of OGTT among participants was 79.4%.
• A significant percentage of pregnant women (20.6%) refused to do OGTT.
• OGTT acceptability was higher among pregnant women with university graduate.
• Two-step approach in the diagnosis of GDM was increasing the rejection rate.
• Among the reasons for OGTT rejection, the media had a significant influence.

The number of studies investigating behaviors of pregnant women to do OGTT are limited in the literature [20–23]. There is some data about this issue from Turkey. These studies report that refusal rate of OGTT for GDM were increasing [24–27]. Rejection of OGTT reduces the chance of GDM diagnosis. Hence, GDM-related risks increase in undiagnosed pregnant women.

It is thought that there is not enough data about the frequency of acceptance of OGTT in Turkish pregnant women. The aim of this study was to investigate the frequency of acceptance of OGTT among participants in our single tertiary center and investigate the possible causes.

## Materials and Methods

This study was conducted at a single tertiary hospital, which is located in Istanbul. The data of non-diabetic 344 pregnant women seen at the Obstetrics Clinic of our hospital between September 2016 and September 2017 were obtained from the hospital records. Women were previously diagnosed with impaired glucose tolerance, and those who did not have regular follow-up during pregnancy were excluded from the study. There were 223 subjects eligible for the study. The study was conducted in accordance with the Helsinki Declaration. All procedures in this study were approved by the Yeditepe University Clinical Research Ethics Committee (date: April 21, 2016, no: 597).

The policy of our Obstetrics Department is to recommend OGTT for all the patients. One of the two or one-step approaches was used in the diagnosis of GDM depending on the choice of the physician following the patient. Two-step approach; non-fasting 50-g 1-h oral glucose challenge test (GCT), plasma glucose concentration with a 140 mg/dL (7.8 mmol/L) cut-off point 1 h after giving 50 g glucose, then a diagnostic 100-g 3-h OGTT was performed after 8–12 h overnight fasting which requires at least two abnormal glucose values. The 100-g 3-h test have been based on the C and C criteria: fasting ≥95 mg/dL (5.3 mmol/L), 1-h ≥180 mg/dL (10.0 mmol/L), 2-h ≥155 mg/dL (8.6 mmol/L) and 3-h ≥140 mg/dL (7.8 mmol/L) [5]. One-step approach; 75-g 2-h OGTT; glucose thresholds were accepted as ≥92 mg/dL (≥5.1 mmol/L) at 0 min (fasting), ≥180 mg/dL (≥10 mmol/L) at the 1^st^ h, and ≥153 mg/dL (8.5 mmol/L) at the 2^nd^ h based on the IADPSG criteria which require at least one abnormal glucose value [6].

Maternal age, gestational age of the OGTT, parity, prepregnancy body mass index (BMI), weight gain during pregnancy, place of residence, comorbid conditions (PCOS, family history of diabetes, history of preterm birth, macrosomic birth, history of GDM, history of gestational hypertension, pre-eclampsia, fetal anomaly, history of stillbirth, history of miscarriage), and outcomes [oligohydramnios, polyhydramnios, small gestational age, large gestational age, preterm delivery, normal vaginal delivery, and cesarean] were obtained from the hospital records. Pregnant women were reached by telephone and asked about some sociodemographic data including working status, educational level, and reasons of the OGTT rejection.

### Statistical Analysis

IBM SPSS Statistics (Inc., Chicago, IL, USA, version 25) program was used for data analysis. Descriptive statistics as mean, standard deviation, frequency, and percentage were used to show the distribution of the socio-demographic, comorbid conditions, and gestational characteristics of the patients. Comparisons were made using the Chi-square test, Mann-Whitney U test, and Fisher’s Exact test. Chi-square test was applied for the comparison of categorical data. The level of significance was defined as a p<0.05.

## Results

**Figure 1.**
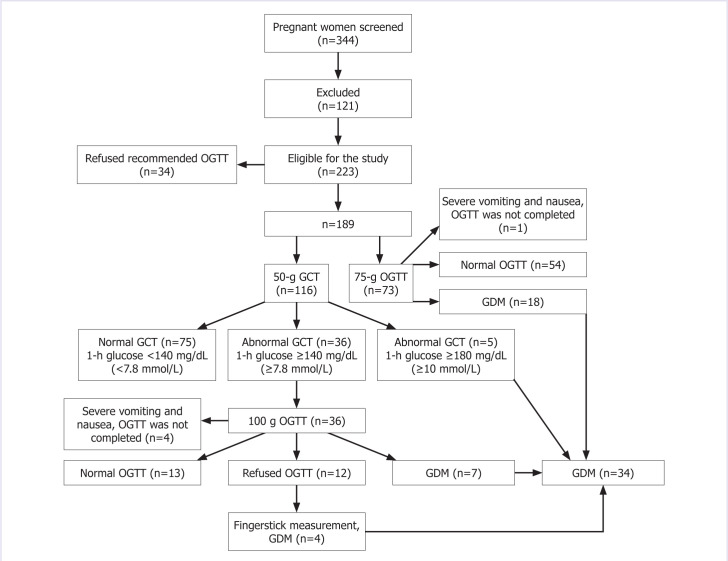
The flow chart of the study. GCT: Glucose challenge test; GDM: Gestational diabetes mellitus; OGTT: Oral glucose tolerance test.

During the study period, 121 out of 344 pregnant women screened for the study who did not have regular follow-up were excluded. The remaining 223 women were included; 34 of the patients never completed the recommended screening test. Finally, 189 of the patients were analyzed for GDM. A total of 116 participants (61.4%) underwent screening for GDM by a two-step approach and the remaining 73 participants (38.6%) underwent screening for GDM by a one-step approach. Twelve out of 36 pregnant women who were recommended 100 g OGTT did not complete the test after positive 50 g GCT. Finally, while 177 pregnant women (79.4%) accepted to do OGTT, 46 pregnant women (20.6%) refused. The flow chart of the study is shown in Figure 1. The frequency of GDM was found to be 15.2% (n=34).

In the total group; the mean maternal age was 31.6±4.9 years, the mean gestational age at OGTT was 25.4±1.4 weeks, the mean pre-pregnancy BMI was 23.6±3.5 kg/m^2^, and the mean weight gain during pregnancy was 14±4.7 kg. Their median parity was 4, ranging from 1 to 9. The baseline characteristics of the subjects are listed in Table 1.

**Table 1.** Baseline characteristics of the patients

Variables	Whole group (n=223)
Maternal age (years) (mean±SD)	31.6±4.9
Gestational age of the OGTT (week) (mean±SD)	25.4±1.4
OGTT accaptence (%)	
Yes	79.4
No	20.6
Prepregnancy BMI (kg/m^2^), (%)	
Underweight, <18.5	5.4
Normal, 18.5–24.9	64.1
Overweight, 25–29.9	20.6
Obese, ≥30	9.9
Prepegnancy BMI (kg/m^2^) (mean±SD)	23.6±3.5
Weight gain during pregnancy (kg) (mean±SD)	14±4.7
Parity, median (min–max)	4 (1–9)
Parity (%)	
1	57.4
2	35.4
≥3	7.2
Working status (%)	
Housewife	15.2
Offical	58.8
Worker	26.0
Educational status (%)	
Primary school+secondary school	1.8
High school	41.7
University	56.5
Place of residence (%)	
Residing out Istanbul	1.8
Hypothyroidism (%)	18.9
Comorbid conditions (%)	
PCOS	2.2
Family history of diabetes	9.4
History of preterm birth	0.4
Macrosomic birth	1.3
History of GDM	1.3
History of gestational hypertension	4.5
Pre-eclampsia	0.9
Fetal anomaly	1.8
History of stillbirth	1.3
History of miscarriage	14.3
Outcomes (%)	
Oligohydramnios	6.3
Polyhydramnios	3.1
SGA	5.4
LGA	8.0
Preterm delivery	6.3
Normal vaginal delivery	34.5
Cesarean	65.5

SD: Standard deviation; GDM: Gestational diabetes mellitus; BMI: Body mass index; OGTT: Oral glucose tolerance test; PCOS: Polycystic ovarian syndrome; SGA: Small gestational age; LGA: Large gestational age.

In the whole study population; 64.1% (n=143) of them were in normal weight group, 57.4% (n=128) of them had first pregnancy, 58.8% (n=131) of them were working as offical workers, 56.5% (n=126) of them had university educational status, and finally, cesarean delivery rate was 65.5% (n=146). The most common accompanying endocrinopathy was hypothyroidism (n=42). When the pregnant women were compared according to OGTT acceptance, there was no statistical significance in terms of variables between the two groups except educational status (Table 2). University graduates were statistically significantly higher in the group that accepted the test (p=0.02). Adverse pregnancy outcomes were similar between the accepting and rejecting groups.

**Table 2.** Comparison of the pregnant women according to OGTT acceptance and rejection

Variables	Accepted OGTT (n=177)	Refused OGTT (n=46)	p
Maternal age (years) (mean±SD)	31.3±4.8	32.9±5.1	0.08*
Gestational age of the OGTT (week) (mean±SD)	25.4±1.4	25.5±1.5	0.98*
Prepregnancy BMI (kg/m^2^) (%)			0.39**
Underweight, <18.5	100	0	
Normal, 18.5–24.9	79	21	
Overweight, 25–29.9	78.2	21.8	
Obese, ≥30	72.8	27.2	
Prepegnancy BMI (kg/m^2^) (mean±SD)	23.4±3.4	24.4±3.8	0.16*
Weight gain during pregnancy (kg) (mean±SD)	14±4.8	14.6±4.3	0.44*
Parity, (%)			0.52**
1	82	18	
2	75.9	24.1	
≥3	75	25	
Parity (mean±SD)	1.5±0.9	1.6±0.7	0.26*
Working status (%)			0.64**
Housewife	85.3	14.7	
Offical	78.6	21.4	
Worker	77.6	22.4	
Educational status (%)			0.02**
Primary-secondary school	25	75	
High school	78.5	21.5	
University	81.7	18.3	
Place of residence (%)			
Residing out Istanbul	75	25	0.60***
Hypothyroidism (%)	76.2	23.8	0.57**
Comorbid conditions (%)			
PCOS	60	40	0.27***
Family history of diabetes	76.2	23.8	0.77***
History of preterm birth	100	0	0.79***
Macrosomic birth	100	0	0.49***
History of GDM	66.7	33.3	0.50***
History of gestational hypertension	70	30	0.45**
Pre-eclampsia	100	0	0.62***
Fetal anomaly	100	0	0.58***
History of stillbirth	66.7	33.3	0.50***
History of miscarriage	81.2	18.8	0.77**
Outcome (%)			
Oligohydramnios	85.7	14.3	0.73**
Polyhydramnios	71.4	28.6	
SGA	75	25	0.91**
LGA	77.8	22.2	
Preterm delivery	64.3	35.7	0.93**
Normal vaginal delivery	84.4	15.6	0.62**
Cesarean	76.8	23.2

SD: Standard deviation; GDM: Gestational diabetes mellitus; BMI: Body mass index; OGTT: Oral glucose tolerance test; PCOS: Polycystic ovarian syndrome; SGA: Small gestational age; LGA: Large gestational age; *: Mann Whitney U test; **: Chi- Square test; ***: Fisher’s Exact test.

When patients were asked about the reasons for OGTT rejection (Table 3); 14 of them (30.4%) thought that it was harmful for the babies and themselves, 21 of them (45.7%) thought that it was unimportant and unnecessary, because of the news they heard from the media, they would check their glucose levels by fingerstick measurements, and prefer to go a diet instead of doing an OGTT, 3 of them (6.5%) said that primary care physicians did not recommend the test, and 1 of them (2.2%) thought that drinking of glucose was too unpleasant.

**Table 3.** Reasons of the OGTT rejection

Reasons	n=46 (%)
Unnecessary-unimportant	45.7
Harmful for me or the baby	30.4
Failed to be contacted	15.2
Doctor did not recommend me	6.5
Drinking of glucose too unpleasant	2.2

OGTT: Oral glucose tolerance test.

## Discussion

In the study population, the majority of the mothers; had normal prepregnancy BMI, had first pregnancy, were working as offical workers, had university educational status, and delivered by cesarean section. We determined that 177 pregnant women (79.4%) accepted to do OGTT, 46 women (20.6%) did not complete at least one OGTT. Thus, we could not determine the actual frequency of GDM. When the pregnant women were compared according to OGTT acceptance, university graduates were statistically significantly higher in the group that accepted the test. The most common reason for OGTT rejection was to find the test unnecessary or harmful due to media influence. The difference of our study from other similar ones is that we report adverse pregnancy outcomes. We also used both one or two-step approaches in the diagnosis of GDM and saw that the two-step approach was increasing the rejection rate.

There is no consensus on which strategy is best for the diagnosis of GDM. One-step approach with IADPSG criteria was associated with significantly higher incidence of GDM and significantly better maternal and perinatal outcomes [28]. The two-step screening test might miss 25% of cases [29]. A study from Turkey found that the prevalence of GDM was 11.1% by IADPSG criteria but 4.48% by C and C criteria [30]. Therefore, IADPSG criteria has gained popularity in recent years, however, there is still controversy about this issue. In our present study, two-step approach and C and C criteria were used in the majority of the group according to the preference of the physician following the patient.

The prevalence of GDM is increasing globally, as well as in Turkey [9]. The prevalence of GDM was 11.5% in Asian population [12], 14% in African countries [13], 5.4% in European countries [31], and 7.6% in U.S [32]. Recently, a national study from Turkey reported that the prevalence of GDM was 16.2% [16]. According to our results, GDM frequency was determined to be 15.2% among participants, however, we could not determine the frequency exactly as the rate of OGTT rejection was high.

A single-center study from Turkey reported that 40% of the women presenting to the center undertook OGTT in 2014, however, this rate was only 12% in 2018 [24]. The other study from Turkey reported that from the second half of 2014, the frequency of application of glucose loading test statistically significantly decreased [27]. Lachmann et al. [21] determined that 12.7% of women (n=242) did not complete at least one OGTT, of whom 32.2% (n=78) never completed testing in a cohort of 1906 women attending a tertiary UK obstetrics center. Our study population was smaller than the UK study, but the percentage of patients who never completed the test or did not complete at least one test was higher in our group. Unlike the studies conducted in this field in our country, our patient recruitment period was longer and all pregnant women had outcomes.

It is known that there is no scientifically confirmed complication associated with the OGTT, except for gastric irritation and delayed gastric emptying [22, 33]. Whilst this does not withhold the patients from refusing to do the OGTT. Previous studies have identified several reasons for the refusal to the OGTT, among those are having nausea due to drinking of glucose load, thinking that OGTT is harmful for the pregnant woman and the baby, not being able to reach the hospital easily to do the OGTT [20–22, 25, 34].

Several authors reported that approximately half of women screened experience nausea during the OGTT [20]. Nausea and vomiting associated with drinking the glucose load after an overnight fast was the most commonly reason for not completing the OGTT in a British population [21]. Another study from The United Arab Emirates reported that vomiting was the major reason for the failure of the OGTT in pregnant women [22]. In our study, there were five patients who could not complete the test due to severe nausea. Four out of 5 patients were in the 100 g group. Based on the results of our study, recommending one-stage test may be considered as an option to avoid this side effect.

The other reasons for non-completion mentioned by the British group were social/mental health issues, and difficulty keeping up with multiple antenatal appointments, due to transport issues or coinciding with other appointments [21]. In an Irish population, the main cause for low uptake rates for GDM screening was the travel distance to screening hospital site [3]. In our cohort, only 4 patients lived outside of Istanbul, however, three of them accepted to do test.

A study from Turkey found that the most frequently reason why some pregnant women refused OGTT was that they thought OGTT was harmful for themselves or the babies [25]. News in the media may have effect on pregnant women about refusing or being hesitant to do the OGTT. Hussain et al. [23] determined that only 17.5% of Malaysian pregnant women living in the rural area were fully aware of the consequences of GDM. In this population, the sources of awareness of GDM were reported to be generally the media, neighbors/friends, and family members. Health-care professionals were sources of information among a lesser proportion of women. This seems to hold true for our population as well. Turkish women are generally affected by a famous Turkish doctor, who claimed in the media that ‘the OGTT is poisoning babies’ and it should not be done to pregnant women [35]. Other reasons claimed were that the pregnant women thought it was unneeded [25]. There were similar reasons in our group; most women stated that it was unimportant and unnecessary, they would prefer to go on a diet instead of doing an OGTT or check their glucose levels by fingerstick measurements, some others found it was harmful to themselves or the babies. This result is thought to be the influence of the media.

It is an important point that pregnant women should have knowledge about the results of GDM. Some authors found that the level of awareness was significantly higher among those with a history of pregnancy than among those who had never been pregnant [24, 26]. Whereas, higher parity was identified as a risk factor for non-completion the OGTT [21, 34]. Parous women (for parity ≥2) were less likely to accept test, since they claimed that they were busy due to childcare issues [21]. Younger maternal age was found to be one of the risk factors related to deny the test [21, 24]. However, age was not found to be significantly associated with the level of knowledge of women about GDM in a Malaysian population [23]. We did not find a significant relationship between the acceptability of the test and age or number of pregnancies.

Educational status was another factor identified as a risk for noncompliance with OGTT. Education level was not found to be significantly associated with the level of knowledge of women about GDM in the Malaysian population [23]. In contrast, current study from Turkey reported that the rate of having OGTT done increased in parallel with higher educational level in Turkish subjects like our study [26].

Considering the risk factors for GDM, women who had a family history of diabetes, whose BMI was ≥30 kg/m^2^ were significantly less likely to complete test [21]. This may relate to feel under surveillance, fears of shaming, or judgment [21]. On the other hand, the Irish data showed that pregnant women who had risk factors for GDM were more likely to attend for screening test [34]. We did not find a significant relationship between test acceptability and risk factors associated with GDM but the number of patients with risk factors associated with GDM was small in our study.

There have been solutions put forward in order to increase the compliance with screening for OGTT. There have been several publications about substituting glucose with food, such as ice-cream or muffin and these have all found correlations of food, with the standard OGTT with glucose load [36, 37]. The intravenous OGTT has been used as an alternative in women experiencing nausea, but this approach has not been validated as well [38]. Also there have been groups who have suggested alternative ways of diagnosis, like using fingerstick measurements [39–41] or fasting plasma glucose measurements [42]. In our study, 4 patients were diagnosed with GDM by using fingerstick measurements. On the other hand, there is data indicating that GCT is a better method than fasting plasma glucose [43].

### Conclusions

In conclusion, the results of the study indicate that refusal rate of OGTT was high in Turkish pregnant women possibly as a result of being influenced by news from media. We believe that pregnant women should be educated about understanding the benefits of OGTT. Another solution may be recommending only the one-step test for pregnant women in countries where the refusal rate of OGTT is high. Based on the results of our study, we switched to the one-step approach to the diagnosis of GDM at our single center.

## References

[1] Kim C, Newton KM, Knopp RH (2002). Gestational diabetes and the incidence of type 2 diabetes: a systematic review.. Diabetes Care.

[2] Hadden DR (1998). A historical perspective on gestational diabetes.. Diabetes Care.

[3] Retnakaran R, Qi Y, Connelly PW, Sermer M, Zinman B, Hanley AJ (2010). Glucose intolerance in pregnancy and postpartum risk of metabolic syndrome in young women.. J Clin Endocrinol Metab.

[4] Petry CJ (2010). Gestational diabetes: risk factors and recent advances in its genetics and treatment.. Br J Nutr.

[5] American Diabetes Association (2020). 2. classification and diagnosis of diabetes: Standards of Medical Care in Diabetes-2020.. Diabetes Care.

[6] Metzger BE, Gabbe SG, Persson B, Buchanan TA, Catalano PA, International Association of Diabetes and Pregnancy Study Groups Consensus Panel (2010). International association of diabetes and pregnancy study groups recommendations on the diagnosis and classification of hyperglycemia in pregnancy.. Diabetes Care.

[7] World Health Organization. (2022). Diagnostic criteria and classification of hyperglycemia first detected in pregnancy; 2013.. https://apps.who.int/iris/handle/10665/85975.

[8] Turkey Endocrinology and Metabolism Society Study and Training Group. (2020). Diabetes Mellitus, and Complications, Diagnosis, Treatment and Follow-up Guide..

[9] Satman I, Omer B, Tutuncu Y, Kalaca S, Gedik S, Dinccag N, TURDEP-II Study Group (2013). Twelve-year trends in the prevalence and risk factors of diabetes and prediabetes in Turkish adults.. Eur J Epidemiol.

[10] Cade TJ, Polyakov A, Brennecke SP (2019). Implications of the introduction of new criteria for the diagnosis of gestational diabetes: a health outcome and cost of care analysis.. BMJ Open.

[11] Sexton H, Heal C, Banks J, Braniff K (2018). Impact of new diagnostic criteria for gestational diabetes.. J Obstet Gynaecol Res.

[12] Lee KW, Ching SM, Ramachandran V, Yee A, Hoo FK, Chia YC (2018). Prevalence and risk factors of gestational diabetes mellitus in Asia: a systematic review and meta-analysis.. BMC Pregnancy Childbirth.

[13] Mwanri AW, Kinabo J, Ramaiya K, Feskens EJ (2015). Gestational diabetes mellitus in sub-Saharan Africa: systematic review and metaregression on prevalence and risk factors.. Trop Med Int Health.

[14] Karcaaltincaba D, Kandemir O, Yalvac S, Güvendag-Guven S, Haberal A (2009). Prevalence of gestational diabetes mellitus and gestational impaired glucose tolerance in pregnant women evaluated by National Diabetes Data Group and Carpenter and Coustan criteria.. Int J Gynaecol Obstet.

[15] Erem C, Kuzu UB, Deger O, Can G (2015). Prevalence of gestational diabetes mellitus and associated risk factors in Turkish women: the Trabzon GDM Study.. Arch Med Sci.

[16] Aydın H, Çelik Ö, Yazıcı D, Altunok Ç, Tarçın Ö, Deyneli O, TURGEP Study Group (2019). Prevalence and predictors of gestational diabetes mellitus: a nationwide multicentre prospective study.. Diabet Med.

[17] Feldman RK, Tieu RS, Yasumura L (2016). Gestational diabetes screening: The International Association of the Diabetes and Pregnancy Study Groups compared with Carpenter-Coustan screening.. Obstet Gynecol.

[18] Guo X, Zheng L, Zhang X, Zou L, Li J, Sun Z (2012). The prevalence and heterogeneity of prehypertension: a meta-analysis and meta-regression of published literature worldwide.. Cardiovasc J Afr.

[19] Agarwal MM, Dhatt GS, Othman Y (2015). Gestational diabetes: differences between the current international diagnostic criteria and implications of switching to IADPSG.. J Diabetes Complications.

[20] Gayet-Ageron A, Poncet B, Guerre P, Rocher L, Dureau-Drevard E, Colin C (2008). Specific information about the WHO guidelines for gestational diabetes screening improves clinical practices.. J Eval Clin Pract.

[21] Lachmann EH, Fox RA, Dennison RA, Usher-Smith JA, Meek CL, Aiken CE (2020). Barriers to completing oral glucose tolerance testing in women at risk of gestational diabetes.. Diabet Med.

[22] Agarwal MM, Punnose J, Dhatt GS (2004). Gestational diabetes: problems associated with the oral glucose tolerance test.. Diabetes Res Clin Pract.

[23] Hussain Z, Yusoff ZM, Sulaiman SA (2015). Evaluation of knowledge regarding gestational diabetes mellitus and its association with glycaemic level: A Malaysian study.. Prim Care Diabetes.

[24] Özceylan G, Toprak D (2020). Effects of controversial statements on social media regarding the oral glucose tolerance testing on pregnant women in Turkey.. AIMS Public Health.

[25] Hocaoglu M, Turgut A, Guzin K, Yardimci OD, Gunay T, Bor ED (2018). Why some pregnant women refuse glucose challenge test? Turkish pregnant women’s perspectives for gestational diabetes mellitus screening.. North Clin Istanb.

[26] Türkyılmaz E, Kelestemur E, Karatas Eray I, Ocal FD, Yavuz Avsar AF (2016). Knowledge level, attitude and behaviours about glucose challenge test among Turkish pregnant women.. Ankara Med J.

[27] Karasu Y (2018). What happened to the glucose loading test? The impact of media on public health.. Med J Ankara Tr Res Hosp.

[28] Badakhsh M, Daneshi F, Abavisani M, Rafiemanesh H, Bouya S, Sheyback M (2019). Prevalence of gestational diabetes mellitus in Eastern Mediterranean region: a systematic review and meta-analysis.. Endocrine.

[29] van Leeuwen M, Louwerse MD, Opmeer BC, Limpens J, Serlie MJ, Reitsma JB (2012). Glucose challenge test for detecting gestational diabetes mellitus: a systematic review.. BJOG.

[30] Karcaaltincaba D, Calis P, Ocal N, Ozek A, Altug Inan M, Bayram M (2017). Prevalence of gestational diabetes mellitus evaluated by universal screening with a 75-g, 2-hour oral glucose tolerance test and IADPSG criteria.. Int J Gynaecol Obstet.

[31] Eades CE, Cameron DM, Evans JMM (2017). Prevalence of gestational diabetes mellitus in Europe: A meta-analysis.. Diabetes Res Clin Pract.

[32] Casagrande SS, Linder B, Cowie CC (2018). Prevalence of gestational diabetes and subsequent Type 2 diabetes among U.S. women.. Diabetes Res Clin Pract.

[33] Fachnie JD, Whitehouse FW, McGrath Z (1988). Vomiting during OGTT in third trimester of pregnancy.. Diabetes Care.

[34] Cullinan J, Gillespie P, Owens L, Dunne F, ATLANTIC DIP Collaborators (2012). Accessibility and screening uptake rates for gestational diabetes mellitus in Ireland.. Health Place.

[35] (2022). https://www.haberturk.com/saglik/haber/1012921-anne-karnindaki-bebegi-zehirliyorsunuz.

[36] Chanprasertpinyo W, Bhirommuang N, Surawattanawiset T, Tangsermwong T, Phanachet P, Sriphrapradang C (2017). Using ice cream for diagnosis of diabetes mellitus and impaired glucose tolerance: an alternative to the oral glucose tolerance test.. Am J Med Sci.

[37] Traub ML, Jain A, Maslow BS, Pal L, Stein DT, Santoro N (2012). The “muffin test”--an alternative to the oral glucose tolerance test for detecting impaired glucose tolerance.. Menopause.

[38] Kim C, Liu T, Valdez R, Beckles GL (2009). Does frank diabetes in first-degree relatives of a pregnant woman affect the likelihood of her developing gestational diabetes mellitus or nongestational diabetes?. Am J Obstet Gynecol.

[39] Dunseath GJ, Bright D, Jones C, Dowrick S, Cheung WY, Luzio SD (2019). Performance evaluation of a self-administered home oral glucose tolerance test kit in a controlled clinical research setting.. Diabet Med.

[40] Allard C, Sahyouni E, Menard J, Houde G, Pesant MH, Perron P (2015). Gestational diabetes mellitus identification based on self-monitoring of blood glucose.. Can J Diabetes.

[41] Ardilouze A, Bouchard P, Hivert MF, Simard C, Allard C, Garant MP (2019). Self-monitoring of blood glucose: a complementary method beyond the oral glucose tolerance test to identify hyperglycemia during pregnancy.. Can J Diabetes.

[42] Rasmussen SS, Glümer C, Sandbaek A, Lauritzen T, Carstensen B, Borch-Johnsen K (2008). Short-term reproducibility of impaired fasting glycaemia, impaired glucose tolerance and diabetes The ADDITION study, DK.. Diabetes Res Clin Pract.

[43] Donovan L, Hartling L, Muise M, Guthrie A, Vandermeer B, Dryden DM (2013). Screening tests for gestational diabetes: a systematic review for the U.S.. Preventive Services Task Force. Ann Intern Med.

